# Three-Electron Dynamics of the Interparticle Coulombic Decay in Doubly Excited Clusters with One-Dimensional Continuum Confinement

**DOI:** 10.3390/molecules27248713

**Published:** 2022-12-09

**Authors:** Joana-Lysiane Schäfer, Fabian Langkabel, Annika Bande

**Affiliations:** 1 Helmholtz-Zentrum Berlin für Materialien und Energie GmbH, Hahn-Meitner-Platz 1, 14109 Berlin, Germany; 2 Institute of Chemistry and Biochemistry, Freie Universität Berlin, Arnimallee 22, 14195 Berlin, Germany

**Keywords:** interatomic Coulombic decay, electron dynamics, quantum dots, continuum confinement, Coulomb barrier

## Abstract

A detailed analysis of the electronic structure and decay dynamics in a symmetric system with three electrons in three linearly aligned binding sites representing quantum dots (QDs) is given. The two outer *A* QDs are two-level potentials and can act as (virtual) photon emitters, whereas the central *B* QD can be ionized from its one level into a continuum confined on the QD axis upon absorbing virtual photons in the inter-Coulombic decay (ICD) process. Two scenarios in such an ABA array are explored. One ICD process is from a singly excited resonance state, whose decay releasing one virtual photon we find superimposed with resonance energy transfer among both *A* QDs. Moreover, the decay-process manifold for a doubly excited (DE) resonance is explored, in which collective ICD among all three sites and excited ICD among the outer QDs engage. Rates for all processes are found to be extremely low, although ICD rates with two neighbors are predicted to double compared to ICD among two sites only. The slowing is caused by Coulomb barriers imposed from ground or excited state electrons in the *A* sites. Outliers occur on the one hand at short distances, where the charge transfer among QDs mixes the possible decay pathways. On the other hand, we discovered a shape resonance-enhanced DE-ICD pathway, in which an excited and localized $B^{*}$ shape resonance state forms, which is able to decay quickly into the final ICD continuum.

## 1. Introduction

The inter-Coulombic decay process (ICD) transforms energy of an inner valence excited or ionized atom (*A*) into kinetic energy of an electron ionized from a nearby other atom (*B*) [1]. The initial state is a Feshbach resonance state [2], delocalized over both atoms, which decays by the two-electron rearrangement. In the past 25 years, ICD was observed and/or theoretically predicted for many different electronic systems, including noble gas clusters [3,4], molecular ensembles [1], biomolecules [5], fullerenes [6,7], and quantum dots (QDs) [8,9], where in the latter the hole level is not necessarily the inner-valence state.

Effectively, ICD is a radiationless energy-transfer (ET) process, which is mediated by the Coulomb interaction among the two involved partnering sites’ electrons, from which the ICD rate is deduced. This resembles the Förster resonance energy transfer (FRET) among chromophores [10], but leads as a surplus not to a final bound but to a final continuum state. Both processes, nonetheless, can be recast into coupled dipole transitions on either site. In this sense, they nail down an asymptotic distance dependence of the rate via the inverse sixth power of the distance between photon donor and acceptor, as was formulated individually for the specific conditions of FRET [11] and of ICD [12,13].

Scientific intuition and simple rationalization suggest clearly that ICD must depend on several more characteristics of the full chemical systems rather than only particle distance, all the more so the less pointlike the acceptor and donor become, as has been compared extensively for FRET [14]. For ICD, this effect was studied in the context of geometrical changes of QDs as ICD partners [15], but also in the context of polarization effects in adjacent molecules [16]. Moreover, the spatial confinement of the ionization continuum to two [17] or even one dimension [8] was found to cause significant deviations from the predicted ICD rate. Finally, neighboring sites may alter the rate already when they form only a barrier or a temporal electron binding site [18]. A strong rate increase can be observed when neighbors with virtual orbitals stabilize the wave function when being located at short distances from the ICD participants allowing for electronic coupling (superexchange ICD) [19,20,21,22]. However, even for well-separated and electronically decoupled neighbors, it was found that an increasing number (*N*) of neighbors of either *A* [23,24] or *B* [25,26] type makes ICD at least *N* times faster [13,27]. The effect depends on the specific geometric arrangement of the neighbors [26,28,29] as well as on the initially excited state. If, for example, two sites *A* are both excited, they may undergo excited ICD (exICD) between each other [23,30,31,32,33,34] or collective ICD (CICD) together with *B*, requiring multiple simultaneous energy-transfer processes to bring up the ionization energy for *B* [24].

Despite this first characterization of the listed ICD pathways, their occurrence and interplay is still rather unexplored. If, for example, asymptotic formulae were used for the prediction of the decay, each possible channel is treated individually [23,24], whereas the electron dynamics treatment includes the full multitude of decay channels [25,26]. However, electron dynamics calculations were not yet done for the ABA system, which was more intensely studied otherwise. Hence, in this paper we target a linearly aligned ABA systems with an electronic confinement along the alignment direction. Such an example is a model for quantum dots in a nanowire, as may be encountered in quantum networks. Moreover, we distinguish two initial resonances states, a singly excited (SE) and a doubly excited (DE) one and compare which processes occur at what inter-QD distance and how they contribute ICD.

In Section 2.1 the pathways are introduced via asymptotic equations, and in Section 2.2 and Section 3 the model and the electron dynamics treatment is explained. In the result Section 4.2.1 and Section 4.2.2, the rates are shown for the complete processes and for individual subprocesses in comparison in order to explain the unexpected lowering of rates compared to that of the regular two-site ICD process.

## 2. Theory

### 2.1. Pathways of the Inter-Coulombic Decay in a Linear ABA Array

**Figure 1:**
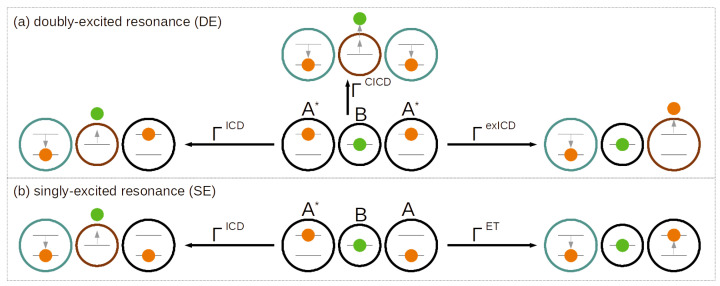
Overview of decay processes of up to three electrons on three Coulomb-coupled sites ABA in linear arrangement. The decaying (**a**) doubly excited (DE) and (**b**) singly excited resonances are shown in the center of each panel. To the left in (**a**) and (**b**) the standard inter-Coulombic decay (ICD) among two sites is shown with relaxation (turquoise) of the electron of site *A* (orange) and ionization (brown) of the electron from *B* (green). To the right, the coupled energy-transfer (ET) among the two sites *A* (orange electrons) is depicted, resulting in an excited-state ICD (exICD) for the DE resonance (**a**) and a Förster-like transfer for the SE resonance (**b**) not leading to ionization. In the DE case, a collective ICD (CICD) through two-photon transfer from relaxation of both *A* (turquoise) can lead to ionization of the central site *B* (brown) as shown along the upward direction.

**Figure 2:**
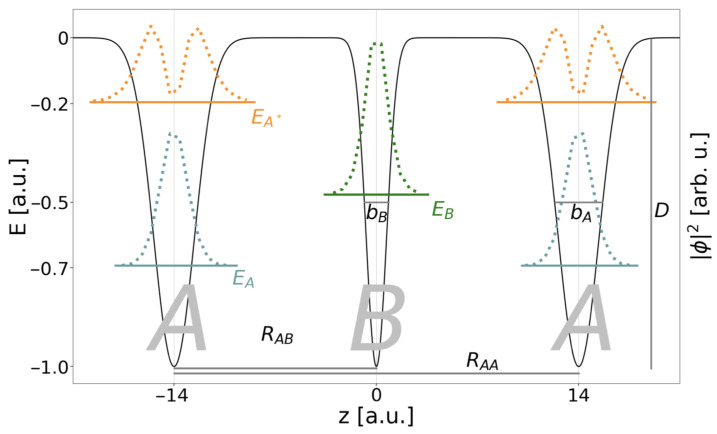
Representation of the three-QD array *ABA* for a distance $R_{AA}=28$ a.u. parametrized according to Table 1. In the negative energy range the single-electron levels *A* (turquoise), *B* (green), and $A^{*}$ (orange) are displayed together with the respective densities ${\left|\phi \right|}^{2}$. Furthermore, the geometric parameters of the binding potential (Equation (9)) are illustrated.

The regular ICD process, an inherent two-electron effect, will occur in its extension by three electrons on three sites along different pathways simultaneously, depending on the underlying electronic structure of the model system. Here, we explicitly focus on a system composed of two two-level sites *A* and one one-level site *B* located exactly in their center as underlying all schematic representations in Figure 1 and Figure 2. The outer sites *A* are separated by the distance $R_{AA}$, whereas the central site *B* in the coordinate origin is distant from each of the other sites *A* by $R_{AB}=R_{AA}/2$. Owing to this arrangement, every two-electron subprocess introduced below has an isoenergetic and symmetry-equivalent counterpart.

The two lowest-energy excited states localized in the array are $A^{*}BA$ and $ABA^{*}$, in which one site *A* is in its excited state. By design of the energetic model it is a Feshbach resonance state [2], which is termed the SE resonance state throughout. With the ICD boundary condition for energies, $\Delta E_{A}>IP_{B}$, imposed, which says that the excitation energy $\Delta E_{A}$ of the *A* site has to be larger than the ionization potential $IP_{B}$ of the *B* site, $A^{*}BA$ can decay into $AB^{+}A$ via regular ICD among only two of the neighbors participating, the third one remaining a spectator. This is sketched in the lower panel (b) of Figure 1 (on the left-hand side) with the relaxing site *A* being encircled in turquoise and the electron-emitting site *B* in brown, according to the persistent color code for this section ignoring for the moment the inactive black site. As said, the process can happen among the central *B* and either of the left- or right-hand side *A*.

The rate $\Gamma^{ICD}$ for a regular two-site ICD process $A^{*}B\to AB^{+}+e^{-}$ in diverse chemical systems was in the past computed through various types of time-independent [35,36,37,38,39,40] as well as time-resolved [8] methods, which prove the validity of a simplified rate equation derived from the Wigner–Weisskopf theory [12,13]. Therein, one electron is assumed to undergo spontaneous radiative decay $A^{*}\to A$ and the other photoionization $B\to B^{+}+e^{-}$. The respective general golden rule ansatz is

(1) $$\Gamma^{ICD}\propto 2\pi |\langle \phi_{1}^{A}\phi_{2}^{B+}|{\hat{r}}_{12}^{-1}|\phi_{1}^{A^{*}}\phi_{2}^{B}{\rangle |}^{2}.$$

In this spin-free ansatz, one assumes separability of the wave function into a product of nonoverlapping single-electron orbital functions ϕ and negligible exchange for the well-separated electrons enumerated 1 and 2, which we will also anticipate for all following derivations of this type. The respective decaying state is coupled by the Coulomb interaction operator ${\hat{r}}_{12}^{-1}$ to a multitude of final continuum states. One core result of Equation (1) for the distance dependence is $\Gamma^{ICD}\propto R_{AB}^{-6}$, which originates from the coupling of the two dipole transitions on the two subunits *A* and *B*. Another is that the rate increases linearly with the number *N* of neighbors [13,27], which will manifest itself in the following discussion.

For the SE decay process, the golden rule ansatz of Equation (1) is extended to three-orbital wave functions [25]. The final state is clearly $\phi_{1}^{A}\phi_{2}^{B+}\phi_{3}^{A}$. On the other hand, the decaying state must be an equal superposition of one excited outer site and one in its ground state, i.e., $2^{-1/2}\left(\phi_{1}^{A^{*}}\phi_{2}^{B}\phi_{3}^{A}+\phi_{1}^{A}\phi_{2}^{B}\phi_{3}^{A^{*}}\right)$. Considering that the Coulomb interaction ${\hat{r}}_{ij}^{-1}$ always couples only two electrons, it allows the factorization of the rate equation into

(2) $$\begin{array}{ccc} \Gamma_{\mathrm{SE}}^{ICD} & \propto & 2\pi |2^{-1/2}\langle \phi_{1}^{A}\phi_{2}^{B^{+}}|{\hat{r}}_{12}^{-1}|\phi_{1}^{A^{*}}\phi_{2}^{B}\rangle \langle \phi_{3}^{A}|\phi_{3}^{A}\rangle \\ & + & 2^{-1/2}\langle \phi_{2}^{B^{+}}\phi_{3}^{A}|{\hat{r}}_{23}^{-1}|\phi_{2}^{B}\phi_{3}^{A^{*}}{\rangle \langle \phi_{1}^{A}|\phi_{1}^{A}\rangle |}^{2}. \end{array}$$

Note that the only terms that are unity are those for which the overlap is kept among the $\phi_{i}$ orbitals factorized from the Coulomb integral (rightmost factor). In the absolute square of the Coulomb integrals (and the prefactors) we identify the two-electron ICD rate of Equation (1); hence

(3) $$\Gamma_{\mathrm{SE}}^{ICD}=2\cdot \Gamma^{ICD}.$$

Beyond the interaction among the *A* and *B* site, a pathway that involves coupling of the two outer *A* sites shall be mentioned. As identical two-level systems, they are candidates for a Förster resonance energy transfer among the electrons depicted in orange in Figure 1b, whereas the green one is spectating [14]. This means that while the excitation on one site decays (turquoise circle), the other site is being excited but not ionized, i.e., $A^{*}A\to AA^{*}$. An ET-rate equation can be set up in the spirit of the ICD rate equation, resulting in

(4) $$\begin{array}{ccc} \Gamma_{\mathrm{SE}}^{ET} & \propto & 2\pi |2^{-1/2}\langle \phi_{1}^{A^{*}}\phi_{3}^{A}|{\hat{r}}_{13}^{-1}|\phi_{1}^{A}\phi_{3}^{A^{*}}\rangle \langle \phi_{2}^{B}|\phi_{2}^{B}\rangle \\ & + & 2^{-1/2}\langle \phi_{1}^{A}\phi_{3}^{A^{*}}|{\hat{r}}_{13}^{-1}|\phi_{1}^{A^{*}}\phi_{3}^{A}{\rangle \langle \phi_{2}^{B}|\phi_{2}^{B}\rangle |}^{2}. \end{array}$$

According to Förster theory it likewise leads to a proportionality $\Gamma_{\mathrm{SE}}^{ET}\propto R_{AA}^{-6}$ [14]. ET is a reversible process in which at any time $A^{*}$ levels are populated to a constant amount. Therefore, ICD is always likewise possible either with the *A* QD on the one side or the other. Moreover, ET does not lead to ionization, so that the rate of Equation (4) will not integrate into an overall decay rate for the three-electron SE system which thus remains $\Gamma_{\mathrm{SE}}=\Gamma_{\mathrm{SE}}^{ICD}$ following $R_{AB}^{-6}$.

In the upper panel (a) of Figure 1, all decay channels of a DE are collected. Given the single-electron levels available on the three sites, this resonance is $A^{*}BA^{*}$. The regular ICD process among two sites *A* and *B* is available for the SE resonance (to the left); here, keeping one spectating two-level site in its excited states $A^{*}$ thus leads to the symmetry-equivalent final states $A^{*}B^{+}A$ and $AB^{+}A^{*}$. The rate is given through the Wigner–Weisskopf derivation [13,25,26,27] as

(5) $$\begin{array}{ccc} \Gamma_{\mathrm{DE}}^{ICD} & \propto & 2\pi |2^{-1/2}\langle \phi_{2}^{B^{+}}\phi_{3}^{A}|{\hat{r}}_{23}^{-1}|\phi_{2}^{B}\phi_{3}^{A^{*}}\rangle \langle \phi_{1}^{A}|\phi_{1}^{A}\rangle \\ & + & 2^{-1/2}\langle \phi_{1}^{A}\phi_{2}^{B^{+}}|{\hat{r}}_{12}^{-1}|\phi_{1}^{A^{*}}\phi_{2}^{B}{\rangle \langle \phi_{3}^{A}|\phi_{3}^{A}\rangle |}^{2}\\ & = & 2\cdot \Gamma^{ICD}. \end{array}$$

Next, there is also a process based on the Coulomb coupling of the electrons at both sites *A* (orange) as shown toward the right-hand side of Figure 1a. It resembles the resonance energy transfer that had been discussed for the SE decaying state and an ICD process at the same time. In addition, one excitation decays into its ground state $A^{*}\to A$ (turquoise circle). The transferred energy is sufficient to ionize the other site (brown circle) according to $A^{*}\to A^{+}$. The process, which we term here excited ICD to distinguish it from regular ICD, has been formulated before [23]. The rate equation is set up as

(6) $$\begin{array}{ccc} \Gamma_{\mathrm{DE}}^{exICD} & \propto & 2\pi |2^{-1/2}\langle \phi_{1}^{A^{+}}\phi_{3}^{A}|{\hat{r}}_{13}^{-1}|\phi_{1}^{A^{*}}\phi_{3}^{A^{*}}\rangle \langle \phi_{2}^{B}|\phi_{2}^{B}\rangle \\ & + & 2^{-1/2}\langle \phi_{1}^{A}\phi_{3}^{A^{+}}|{\hat{r}}_{13}^{-1}|\phi_{1}^{A^{*}}\phi_{3}^{A^{*}}{\rangle \langle \phi_{2}^{B}|\phi_{2}^{B}\rangle |}^{2} \end{array}$$

based on the fact that there may be two symmetry-equivalent pathways leading to the two final states $ABA^{+}$ and $A^{+}BA$. In terms of the decay behavior, this does not differ from any ICD process with lower exited states, i.e., it obeys the same distance behavior $R_{AA}^{-6}$ as well as other relations which are deduced from the Wigner–Weisskopf rate equation. Note that in a collinear arrangement, the maximal distance among both sites *A*, $R_{AA}=2R_{AB}$, may cause a significantly lower rate $\Gamma^{exICD}<<\Gamma^{ICD}$ nonetheless, whereas some bent arrangements may cause a closer proximity among both *A* than among *A* and *B*, leading thus to a very fast exICD.

Note that the creation of a DE initial state is particular here, and can be achieved, e.g., by a very short [23] or intense pulse [30,31]. There had been a theoretical study on neon dimers undertaken with the Fano–Stieltjes approach, which considers exICD for neon distances shorter than the distance where the asymptotic formula might become valid [23]. It was followed by the derivation of analytical equations of motion for the electron dynamics combined with nuclear dynamics on the excited state potential energy surfaces [30] and ultimately confirmed experimentally in neon dimers [31] also for decay cascades including higher excited neon states in clusters [32]. The exICD was also shown for helium droplets, where it was found to scale with the number of neighbors [33,34].

Much more unexplored are the collective ICD processes [24], in which all electrons participate. In a two-photon energy transfer, the central site *B* is ionized (and excited, superscript +∗) in that both sites *A* deexcite simultaneously, as depicted toward the top in Figure 1a. Note, if *B* was DE into a bound state, the process would be a special form of resonance energy transfer called energy pooling [14].

The Wigner–Weisskopf formulation for the CICD three-electron process based on two-electron interactions uses second-order perturbation theory [24], giving as rate ansatz for our $A^{*}BA^{*}$ example system

(7) $$\Gamma_{\mathrm{DE}}^{CICD}\propto 2\pi \sum_{t}{\left|\frac{\langle \phi_{1}^{A}\phi_{2}^{B^{+*}}\phi_{3}^{A}|{\hat{r}}_{ij}^{-1}|\Phi_{t}\rangle \langle \Phi_{t}|{\hat{r}}_{ij}^{-1}|\phi_{1}^{A^{*}}\phi_{2}^{B}\phi_{1}^{A^{*}}\rangle }{E_{A^{*}BA^{*}}-E_{t}}\right|}^{2}.$$

Here, the transitions of the three electrons are split into virtual two-photon processes with different intermediate configurations *t*. Those can be either the state resulting from two *A* relaxations, $2A^{*}\to 2A$, the state after the *B* ionization with two photons, $B\to B^{+*}+e^{-}$, or the states after a regular or excited ICD process, i.e., one state out of ABA, $A^{*}B^{+*}A^{*}$, $2^{-1/2}\left(AB^{+}A^{*}+A^{*}B^{+}A\right)$ or $2^{-1/2}\left(AB+BA\right)$. No matter which one is chosen, both Coulomb integrals in our QD formulation give a dependency $R^{-3}$ for the dipole–dipole transition in the short-range resonance-energy transfer regime [14] applicable to the distance and transferred energies encountered in the ABA system. As the two integrals in Equation (7) multiply and are being squared, the rate for CICD follows $R_{AA}^{-12}$. However, with $R_{AA}^{-12}$ the rate $\Gamma^{CICD}$ decreases much more quickly than that of regular ICD, making CICD generally noncompetitive at long distances. Hence, CICD could only be seen under rigorous energy constraints excluding regular and excited ICD. This can be rationalized by being an unlikely three-particle process [23]. Only at short distances might it dominate other decay channels, but for such cases, Fano–ADC calculations on Kr${}_{2}$Ar clusters resulted in lower rates than were predicted by the asymptotic formula [24]. Note that in the first work on CICD on Kr${}_{2}$Ar clusters, the authors have assumed one of the interatomic distances to be as large as the wavelengths of the transferred photon (approximately 100 nm) and hence one integral obey $R^{-2}$ [24].

Conclusively, with three contributions, the overall rate for DE-ICD, $\Gamma^{\mathrm{DE}}=\Gamma_{\mathrm{DE}}^{ICD}+\Gamma^{exICD}+\Gamma^{CICD}$, is richer than for SE, where we can, however, expect a lowering importance of contributions from left to right. For the dominating rate $\Gamma_{\mathrm{DE}}^{ICD}$, a rate doubling is expected with an additional rate increase of the latter terms.

Moreover, any other decay processes can be largely excluded for the underlying model. The occupation of each few-level site with only a single electron as well as the energetics within the system exclude the occurrence of an Auger–Meitner process [41,42], to which ICD has to be compared in core-excited or ionized atoms and molecules. Then, we exclude any nuclear motion of the atoms forming one site. In cases of the sites being atoms or small molecules, instead, the nuclear motion was found to lead to fluctuating ICD rates [4,43,44,45,46,47]. For the sites being quantum dots, they would not move with respect to one another but rather, internally. However, such phonon-mediated dissipation was found to not compete with ICD unless their distances become very large [48]. Finally, the most straightforward radiative decay of the excited state $A^{*}$ is known to be significantly slower than the discussed energy-transfer processes for any of the studied ICD materials [8].

### 2.2. Electron Dynamics in Model Potentials

The purpose of this study is to investigate the interplay of several simultaneously available ICD and related processes’ channels in the context of fully correlated electron-dynamics computations. For computational feasibility and for some freedom in designing a few-level electronic structure, model potentials are used to reflect the three electron-binding sites ABA. Furthermore, this arrangement allows us to deliberately remove the spectator electron and its binding site for the discussed two-electron subprocesses, so that we target the role of the respective spectator electron site with those two, which are active participators in the process. In particular we can also reformulate the model into a single-electron picture for the ICD electron, setting up effective potentials imposed by neighboring sites and electrons, which is another means for interpretation of the full three-electron dynamics observed.

The specific potentials displayed in Figure 2 are models for quantum dots in a nanowire [8,25], in which the electronic motion occurs in one dimension along the *z* direction only, such that the two other Cartesian coordinates can be omitted [49]. The respective one-dimensional electronic Hamiltonian in atomic units for *N* electrons and *M* QDs reads

(8) $$\hat{H}=\sum_{i=1}^{N}\left(-\frac{1}{2}\nabla_{z_{i}}^{2}+\sum_{k=1}^{M}{\hat{V}}_{k}^{\mathrm{QD}}\left(z_{i}\right)+{\hat{V}}_{\mathrm{CAP}}\left(z_{i}\right)+\sum_{j<i}^{N}{\hat{r}}_{ij}^{-1}\right).$$

The summands define the kinetic energy, the QD confinement potential for each QD *k*, as well as the complex absorbing potential (CAP) for each electron *i* and the Coulomb interaction between the two electrons *i* and *j*.

The electronic structures of QD conduction bands open to a nanowire environment are represented by Gaussian potentials shown in Figure 2 and given by

(9) $${\hat{V}}_{k}^{\mathrm{QD}}\left(z_{i}\right)=-D_{k}\exp\left(-b_{k}{\left({\hat{z}}_{i}-z_{k}\right)}^{2}\right).$$

Here, $b_{k}$ relates to the widths of the Gaussian potential and is reproducing the QD extension along the nanowire, and $z_{k}$ marks the position. Throughout this study, the electron-emitting QD *B* with one electronic level is placed in the origin of the *z* axis and is framed by one or two two-level QDs *A* at positions $-R_{AB}$ only or $\pm R_{AB}$. $R_{AA}$ is the distance between the minima of the respective potentials of the *A*-type QDs. $D_{k}$ is finally the depth of the binding potential, and the energetic zero point marks the onset of the continuum for unconfined electrons.

The last single-electron operator of Equation (8) is a CAP with

(10) $${\hat{V}}^{\mathrm{CAP}}\left(z_{i}\right)=-i\left({\hat{W}}_{z}^{L}+{\hat{W}}_{z}^{R}\right).$$

Already anticipating the concepts of electron dynamics introduced below, the CAP hinders a continuum ICD electron wave packet from backscattering onto the QD system at the edges of the finite grid by transferring it into the imaginary space. Actually, two CAP operators

(11) $${\hat{W}}_{z}^{L,R}=\eta \;{\left|z-z_{L,R}\right|}^{n}\;\Theta \left(\pm \left(z-z_{L,R}\right)\right).$$

are placed to the left (*L*) and the right (*R*) side of the QD array along the negative and positive *z* direction, respectively. They are defined through the strength parameter η, the order *n*, the onset position $z_{L,R}$ and the Heaviside step function Θ, which ensures that the CAP vanishes for $|z<z_{L,R}|$.

The Coulomb-interaction operator essentially mediating ICD, is by virtue six-dimensional and nonseparable. Because the two interacting particles are in a one-dimensional model, here an effective Coulomb potential,

(12) $$\hat{V}{\left(z\right)}_{ij}=\sqrt{\frac{\pi }{2}}\exp\left(\frac{z_{ij}^{2}}{2}\right)\mathrm{erfc}\left(\frac{z_{ij}}{\sqrt{2}}\right),$$

is used [49,50]. It is derived for the case of a wire potential with a strong harmonic oscillator confinement in *x* and *y* directions, the excited states of which are energetically inaccessible here, such that the wave function can be separated and *x* and *y* components integrated.

For analysis reasons, we define an effective potential for the ICD electron *j* in *B* [8]. To this end, the electrons *i* occupying single-particle bound states $\phi_{n}\left(z_{i}\right)$ with n=A or $A^{*}$ of the two *A* QDs and their Coulomb repulsion with the *B* electron are added to the general binding potential giving

(13) $${\hat{V}}^{\mathrm{eff}}\left(z_{j}\right)=\sum_{k=1}^{M}{\hat{V}}_{k}^{\mathrm{QD}}\left(z_{i}\right)+\sum_{i=1}^{\left(N-1\right)}\langle \phi_{n}\left(z_{i}\right)|r_{ij}^{-1}|\phi_{n}\left(z_{i}\right)\rangle .$$

In order to execute electron dynamics simulations, the *N*-electron wave packet is given in the antisymmetrized multiconfiguration time-dependent Hartree (MCTDH [51,52]) form

(14) $$\Psi \left(z_{1},\ldots ,z_{N},t\right)=\sum_{j_{1}}^{n_{1}}\ldots \sum_{j_{N}}^{n_{N}}A_{j_{1},\ldots ,j_{N}}\left(t\right)\prod_{\kappa =1}^{N}\varphi_{j_{\kappa }}^{\left(\kappa \right)}\left(z_{\kappa },t\right).$$

The antisymmetry in electron exchange is introduced by a condition on the expansion coefficients,

(15) $$A_{j_{1},\ldots ,j_{l},\ldots ,j_{k},\ldots ,j_{N}}\left(t\right)=-A_{j_{1},\ldots ,j_{k},\ldots ,j_{l},\ldots ,j_{N}}\left(t\right),$$

thus realizing a quartet state for three electrons. Furthermore, a number of $n_{\kappa }$ single-particle functions (SPFs) $\varphi_{j_{\kappa }}^{\left(\kappa \right)}\left(z_{\kappa },t\right)$ for each degree of freedom (DOF) κ (each electron here) is used and expressed in a time-independent basis set as

(16) $$\varphi_{j_{\kappa }}^{\left(\kappa \right)}\left(z_{\kappa },t\right)=\sum_{i_{1}=1}^{N_{\kappa }}c_{i_{\kappa }}^{\kappa ,j_{\kappa }}\left(t\right)\chi_{i_{\kappa }}^{\left(\kappa \right)}\left(z_{\kappa }\right),$$

where $c_{i_{\kappa }}^{\kappa ,j_{\kappa }}\left(t\right)$ are the time-dependent expansion coefficients and $\chi_{i_{\kappa }}^{\left(\kappa \right)}\left(r_{\kappa }\right)$ is a primitive basis function. On the basis level, those are ultimately implemented within a discrete variable representation (DVR) [53,54,55].

MCTDH approximates the solution of the time-dependent Schrödinger equation by using the Dirac–Frenkel variational principle to derive equations of motion for the MCTDH expansion coefficients and SPFs, which are propagated in time.

All desired observables for the interpretation of the dynamical processes of the electrons in the QD systems are obtained from the propagated wave packet. The absolute square of the projection of the time-dependent wave function Ψ(t) onto the initial wave function Ψ(0), i.e., the squared autocorrelation function, gives information about the decay process via the decay rate Γ [8], which is obtained by fitting the exponential slope to

(17) $${\left|a\left(t\right)\right|}^{2}={\left|\langle \Psi \left(0\right)|\Psi \left(t\right)\rangle \right|}^{2}=e^{-\Gamma t}.$$

To analyse the populations of the different single-electron states *s*, a projection

(18) $$P_{s}\left(t\right)=N|\langle \phi_{s}|1^{N}{\left|\Psi \left(t\right)\rangle \right|}^{2}$$

of the time-dependent *N*-electron wave function on the respective one-electron wave function $\phi_{s}$ with $s=A,A^{*},B$ is performed, including a projection on an *N*-electron identity matrix $1^{N}$. For continuum states, we are reintroducing the continuum contribution into their population [56].

## 3. Computational Details

MCTDH calculations are executed with the Heidelberg program [53,57]. A sine DVR in the interval [−100,100] with 300 grid points represents the primitive basis. CAPs are placed at $z_{L,R}=\pm 50$ a.u. The CAP order is set to n=3 and the strength to $\eta =9.5\cdot 10^{-7}$ a.u. Furthermore, the effective Coulomb operator (Equation (13)) is brought into a sum-of-products form by using the potfit subroutine [58].

For block improved relaxations [59,60] in the CAP-free system, which give the eigenstates with discretized continuum, typically $n_{\kappa }=48$ SPFs are used for each mode. In rare cases of numerical instabilities during relaxation, the number of SPFs is increased to at most 80 SPFs. In the propagations $n_{\kappa }=8$ SPFs are sufficient. In both types of MCTDH calculations, a constant mean fields integrator (CMF) is applied with an accuracy of $10^{-2}$ a.u. or $10^{-8}$ a.u. for the relaxation and propagation calculations, respectively. CMF step sizes are fixed to 0.1 a.u. in relaxations and variable in propagations. The SPFs are relaxed (propagated) by using the Runge–Kutta method of order 8 with an error tolerance $10^{-6}$ ($10^{-8}$) a.u. and an initial step size of 0.1 a.u. Improved relaxation furthermore requires a Davidson routine to diagonalize the vector of MCTDH-coefficients, using here a maximal order of 800 and an accuracy of $10^{-6}$ a.u. The one-dimensional initial functions are chosen as Gaussian functions. Their width is 2.0 a.u. To propagate the vector of MCTDH coefficients, the short iterative Lanczos algorithm is used with an order of 15 and a step size of $10^{-8}$ a.u. The total propagation time is chosen differently for the systems (10${}^{4}$–10${}^{5}$ a.u.) to ideally reveal the decay happening at different rates.

The binding potential of the respective QD system is defined in Equation (9), with either two or three QDs, M=2,3. The depth of the respective binding potential is always D=1 a.u. and the sizes of the QDs are chosen to be $b_{A}=0.25$ a.u. and $b_{B}=1.0$ a.u. A scan over the distance between the outer QDs is performed in the interval $R_{AA}=\left[20,70\right]$ a.u.

## 4. Results

### 4.1. Electronic Structure

The present work focuses on the dynamical processes undergone by three electrons in three linearly aligned QDs. Figure 2 depicts the Gaussian binding potential model for the QD array which is designed such that the central QD is of *B* type with one energy level and the outer two of *A* type. The corresponding one-electron energy level values are listed in the two leftmost columns of Table 1. The model was designed such that the energy difference between the two levels on site *A*, $\Delta E_{A}$, is always larger than the ionization energy of *B*, $IP_{B}$. This implies that already only one excited outer electron in a state $A^{*}$ suffices to open the ICD pathway, whereas ET is possible anyway. Likewise, for two excited electrons in two states $A^{*}$ all SE and DE pathways sketched in Figure 1 shall be accessible.

**Figure 3:**
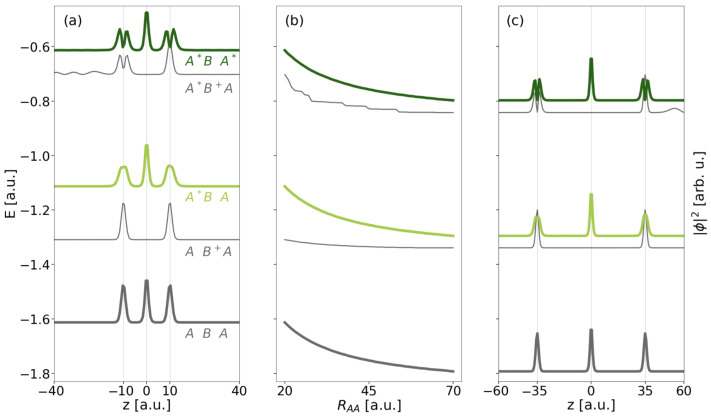
The energies of the key localized states of the three-electron three-QD system are displayed as a function of distance $R_{AA}$ (panel (**b**)). From bottom to top ground state (ABA), first continuum state of type $AB^{+}A$, lower of SE resonances $A^{*}BA$, first continuum state of type $A^{*}B^{+}A$, and DE resonance $A^{*}BA^{*}$ are displayed. The normalized three-electron densities ${\left|\Psi \left(0\right)\right|}^{2}$ in the left- and rightmost panel are leveled by the respective state energies at the given distance $R_{AA}=20$ a.u. in (**a**) and 70 a.u. in (**c**).

An overview of energies and electron densities ${\left|\Psi \left(0\right)\right|}^{2}$ of the three-electron eigenstates with respect to increasing distance $R_{AA}$ between the outer QDs is given in Figure 3 and in Table 1 as obtained from MCTDH relaxation calculations. In panels (a) and (c), corresponding to distances $R_{AA}=20$ and 70 a.u., both localized resonance states of interest can be identified by density inspection. The DE resonance $A^{*}BA^{*}$ depicted as dark green top line has the highest energy listed ($E_{A^{*}BA^{*}}=-0.613$ a.u. for $R_{AA}=20$ a.u.). Its density clearly indicates the even distribution of electrons onto the QD. One electron is in QD *B* occupying its only state and hence showing a Gaussian-type density, while excited states $A^{*}$ of the other two QDs are occupied such that the local density there has a node centered on the QD. Upon increase of the distance $R_{AA}$, the state energy clearly drops due to the significantly decreasing Coulomb interaction of electrons on each pair of sites *A* or *B*.

The SE resonance (light green, third from top), which is twofold, degenerates into $ABA^{*}$ and $A^{*}BA$ serving both as initial states for the two processes presented in Figure 1b at $R_{AA}=20$ a.u. It has with $E_{A^{*}BA}=-1.113$ a.u. a lower energy than the DE resonance by the approximately 0.5 a.u. corresponding to the energy difference among *A* and $A^{*}$, which likewise applies to all shorter distances as well. The local electron density on the outer QDs has a broad and flat peak due to the superposition of the $A^{*}$ and *A* density contributions, whereas the local density contribution on *B* remains unchanged compared to $A^{*}BA^{*}$. The other, degenerate state (not shown) has generally the same density profile. The $E-R_{AA}$ profile (b) follows the same trend as of the DE resonance and likewise does the ground state. It has three localized electrons ABA and $E_{ABA}=-1.613$ a.u. at $R_{AA}=20$ a.u. Both electrons on the *A* side occupy the lower state of the two-level system and have a narrow Gaussian-type density like the electron occupying *B* (thick black bottom line in Figure 3).

Energetically in between the ground state and each of the resonances, there are the onsets of the two series of ICD continua into which the respective DE and SE resonances can decay. For the SE resonance this continuum sets on at $E_{AB^{+}A}=-1.309$ a.u. ($R_{AA}=20$ a.u.). It consists of states of type $AB^{+}A$, meaning that there are two electrons localized in the *A* levels of the outer QDs, whereas no electron resides in the central QD. The third electron establishes density outside the area of the QDs, which is not visible from the representation in Figure 3, because it particularly spreads beyond z=±70 a.u. Compared to the localized states the energy slope (b) is less steep here, because the electron from *B* has moved toward the edge of the grid and is basically not contributing to the Coulomb interaction, which is then mainly composed of interaction of two electrons in both *A* sites of amount $R_{AA}^{-1}$ only.

The other series of continuum states resulting from the ICD of the DE resonance sets has densities revealing the displayed $A^{*}B^{+}A$-type (and nearly isoenergetic inverted $AB^{+}A^{*}$-type states, not shown). Again, the *B* side is not populated, whereas one outer QD is populated in the excited and one in the ground state. As can be seen on the right-hand side of the density in panel (c) and on the left-hand side in panel (a), the emitted electron assembles outside the QD region and also beyond the area shown ([−40,40] a.u.). Note that the contribution of the emitted electron in (a) has nodes for z≤−15 a.u. and sets on energetically at $E_{A^{*}B^{+}A}=-0.702$ a.u. ($R_{AA}=20$ a.u.). As the first continuum states typically has no nodes, here we have certainly not fully converged the continuum in the improved block-relaxation computation. This does not affect the intuitive understanding of the state manifold, but the shape of the $E-R_{AA}$ curve (b), which is not as flat as seen for the other continuum. The propagation is later executed in another functional basis and will therefore not suffer from an inaccurate state representation here.

**Figure 4:**
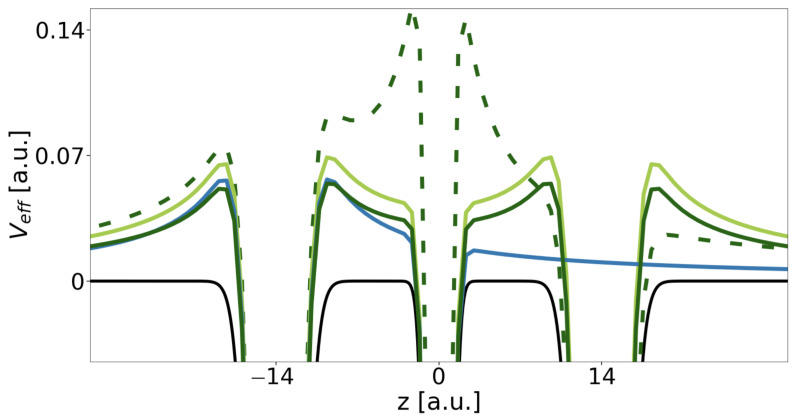
Illustration of the three-QD array *ABA* potential (black) displayed for $R_{AA}=28$ a.u. within [−0.045,0.152] a.u. Furthermore, the effective Coulomb barriers resulting from electron configurations with the electron from *B* in the continuum, i.e., $A^{*}B^{+}A$ (dark green), $AB^{+}A$ (light green) and $AB^{+}$ (blue), respectively, as well as effective Coulomb barriers resulting from excited ICD electron configurations, i.e., $ABA^{+}$ (dashed dark green), are represented.

Although the energy difference between the two initial states for ICD is nearly constant with increasing $R_{AA}$, the energy difference among them and the onset of their ionization continuum decreases. The kinetic energy of the ICD electron decreases likewise. Moreover, for the one-dimensional continuum we have observed effects that depend on the continuum electron’s energy in conjunction with effective repulsive Coulomb barriers established by the remaining bound electrons in their final states *f* [8,17,25,61,62]. The effective potentials (Equation (13)) established for the DE- and the SE-ICD final state are shown in Figure 4 as dark and light green lines relative to the pure binding potential (Equation (9)) in black.

The DE final state (dark green) is a superposition state of $A^{*}B^{+}A$ and $AB^{+}A^{*}$ and shows a maximal barrier height of $E_{B}^{f}=0.054$ a.u. Only when the electron ionized from *B* has sufficient kinetic energy to overcome this barrier can the decay process be expected to occur unhindered, which is the case for all $R_{AB}<17$ a.u. (cf. Table 2). Otherwise situations may occur in which the electron is reflected from the Coulomb barriers and thus might be trapped in between both QDs or where the rate oscillates as a function of $R_{AB}$. For the SE resonance, the barrier height in the final state $AB^{+}A$ is $E_{B}^{f}=0.070$ a.u. It is higher, because an electron in the *A* ground level has a larger contribution to the effective potential compared to an electron in the $A^{*}$ excited level. The ICD electron overcomes the barrier for distances below $R_{AB}=25$ a.u. This distance is larger despite the higher barrier, because the SE resonance is higher above its continuum than the DE resonance (cf. Figure 3). For comparison the two-electron two-QD setup would establish one effective barrier maximum at $V_{\mathrm{eff}}=0.056$ a.u. hindering all electrons with $R_{AB}\geq 15.5$ a.u. Finally, one effective potential is shown for the final state of the exICD of the DE resonance, i.e., $ABA^{+}$ (dark green, dashed). Here, the two remaining electrons establish a huge barrier around the *B* QD of $E_{B}^{f}=0.152$ a.u., however due to the large kinetic energy of the exICD electron not leading to its hindrance within the analyzed range of distances (only for $R_{AB}>35.0$ a.u.).

Ultimately, all single-electron state energies increase within the effective potential, whereby a state in the $AB^{+}A$ potential is higher than in the $A^{*}B^{+}A$ potential, e.g. the energy of the *A* level in the DE potential is $E_{A}\left(R_{AA}=28\right)=-0.599$ a.u. and in the $AB^{+}A$ potential $E_{A}\left(R_{AA}=28\right)=-0.528$ a.u.

### 4.2. Electron Dynamics

In the following, the electron dynamics of the decays of the DE and SE resonances is presented in terms of rates Γ computed from the absolute square of the autocorrelation function (Equation (17)), the norm as function of time, and the transient population of single-electron states (Equation (18)). In addition to the overall three-electron dynamics, a comparison with related two-electron dynamics of subprocesses in all three or only two QD potentials is offered for the DE electron configuration.

**Figure 5:**
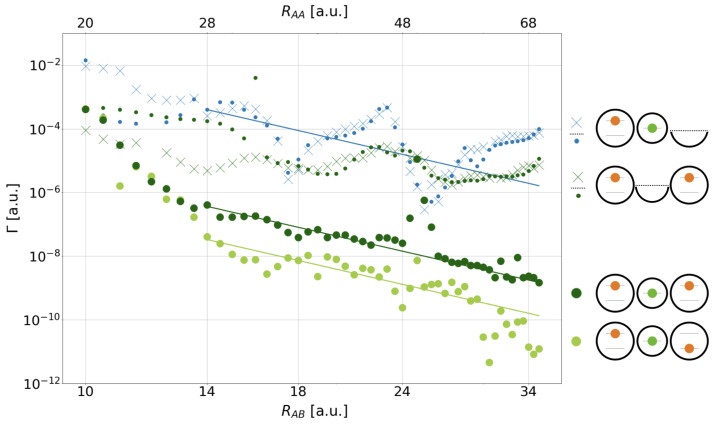
Double-logarithmic representation of the decay rates Γ as function of interdot distance $R_{AB}$ (top abscissa $R_{AA}$) for the decay processes arising from the SE and DE initial electron configuration (light and dark green large dots). The configurations are shown on the right side (bottom) also for related two-electron processes (above) in the order of their decay rates. On top, the two-QD processes (blue, dark green crosses) are shown, followed by the two-electron, three-QD processes (blue, dark green small dots); the empty/removed QD is depicted as a half circle on the right. The asymptotic regime for regular, SE-, and DE-ICD is indicated by the rates’ least-squares fit to $R_{AB}^{-6}$ shown as solid lines in the corresponding color.

Figure 5 collects all decay rates as function of the distance $R_{AA}$ (top abscissa) and $R_{AB}$ (bottom abscissa) in a double-logarithmic representation. As all processes are considered extensions to regular ICD among two electrons on two sites (cf. Figure 1, left), the top $\Gamma -R_{AB}$ curve (blue crosses) applies to this regular ICD among only two electrons, and its sketch is displayed right next to the graph. Furthermore, a solid blue line is the fit of the $R_{AB}^{-6}$ Wigner–Weisskopf asymptote to the data. The rates follow the general asymptotic trend, but oscillate, which was observed likewise for slightly modified QD pairs earlier and can be explained by the Coulomb barrier hindering the free motion of the ICD electron within the one-dimensional continuum; however, they sometimes allowing for tunneling (at highest Γ), leading to an effect beyond three orders of magnitude [8,25,61,62]. Here for paired QDs, the blockade sets on from $R_{AB}>15.5$ a.u. (cf. Table 2). Similar to these former results is the order of magnitude of the average rates, e.g., $10^{-3}$ a.u. at $R_{AB}\approx 12$ a.u. and $10^{-5}$ a.u. at around twice that distance [8,25,61,62]. Note that the other curves of Figure 5 are going to be discussed whenever the respective processes are discussed in the following sections.

#### 4.2.1. Dynamics of the Doubly Excited Resonance

**Figure 6:**
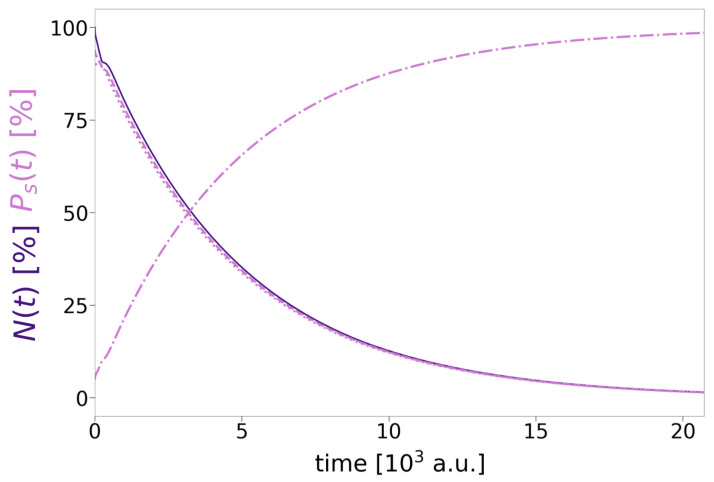
Time propagation of the norm N(t) (dark purple, solid) as well as the single-state populations $P_{s}\left(t\right)$ (light purple) of $A^{*}$ (dashed), *A* (dashed-dotted), and *B* (dotted) in % shown for $R_{AB}=10$ a.u.

The decay of the three-electron three-QD DE resonance $A^{*}BA^{*}$ into the two symmetry-equivalent states $A^{*}B^{+}A$ and $AB^{+}A^{*}$ is the topic of this chapter. The most straightforward means to verify this expected decay is to inspect the level populations $P_{s}\left(t\right)$ in conjunction with the norm N(t) as a function of time. The ones of $R_{AB}=10$ a.u. (Figure 6) exemplify the behaviour for nearly all distances, for which the only distinguishing feature is the increasing duration of the process with distance (very few outliers will be discussed later). After an equilibration time of 300 a.u. for the initial noneigenstate, the decrease of the norm (solid dark-purple line) during propagation is exponential. It follows the decay of the squared autocorrelation function used to deduce the decay rate $\Gamma_{\mathrm{DE}}^{ICD}$ (Equation (17)). The decay comprises the emission of the *B*-type electron (dotted light-purple lines) and its absorption by the CAP along with the relaxation of the $A^{*}$ electron (decreasing dashed line) into the *A* state (increasing dashed-dotted line). The behavior is the same as was observed for any regular two-electron ICD [8,61].

The rate of the DE resonance decay for all studied distances $R_{AB}$ is displayed as dark green bold dots in Figure 5. The graph sets on at $4.63\cdot 10^{-4}$ a.u., two orders below that of regular ICD, and firstly decreases quickly for $10\;a.u.\leq R_{AB}\leq 14\;a.u.$ by nearly three orders of magnitude and then establishes its $R_{AB}^{-6}$ trend within $14\;a.u.\leq R_{AB}\leq 35\;a.u.$, leading in this larger range again to a decrease by more than two orders of magnitude. Two major differences in comparison to the two-electron ICD rate (blue crosses) jump to the eye: on the one hand, the $A^{*}BA^{*}$ decays neatly, but less systematically, and follows the asymptote with only few obvious outliers around 25 a.u. and 32 a.u.

On the other hand, counterintuitively, the rates in the $R_{AB}^{-6}$ regime are all in the range of $\Gamma_{\mathrm{DE}}\approx 10^{-7}$–10${}^{-9}$ a.u. and thus orders of magnitude smaller than the regular two-electron ICD rates of $\Gamma_{\mathrm{DE}}^{ICD}\approx 10^{-3.5}$–10${}^{-5.5}$ a.u. This disproves the original hypothesis $\Gamma_{\mathrm{DE}}=\Gamma_{\mathrm{DE}}^{ICD}+\Gamma^{exICD}+\Gamma^{CICD}$ of Section 2.1 for the one-dimensional continuum model system (cf. Figure 1), which postulated already a speeding according to $\Gamma_{\mathrm{DE}}^{ICD}=2\Gamma^{ICD}$ plus contributions from the expectedly less relevant exICD and CICD processes. Given the trend of rates only, we cannot distinguish exICD with its $R_{AA}^{-6}$ trend from ICD following the same asymptote. The only process we can exclude is CICD, as no trend $R_{AA}^{-12}$, e.g., along a steeper asymptotic slope, is seen in the asymptotic regime in the data points.

For the very low rates, inhibition of the ICD electron by the remaining bound electrons is of greatest importance. Two main profiles arise depending on the number of electrons surrounding the ICD electron. In two-electron systems as the one of regular ICD or the exICD system, the ICD electron populates an outermost QD. The related effective potentials, blue $AB^{+}$ and dashed dark green $ABA^{+}$ in Figure 4, respectively, have side-dependent barrier heights. By contrast, if the ICD electron is emitted from *B* in three linearly aligned QDs, a symmetric barrier is established along both emission directions (dark and light solid green lines), confining the electron from *B*. Here, the motion of the *B* electron is twice as restricted as in the two-electron systems and asymmetric exICD system. The rates are three orders lower. The amount of this lowering derives from the oscillations for regular ICD. The rate maxima (minima) correspond to a decay resulting in the continuum electron on one side (both sides) [8]. In the latter case, hindered electron tunneling through the effective barrier causes ICD slowing by two to three orders of magnitude. The emission of *B* within the three-electron dynamics involves tunneling through the effective barriers on both sides to which the *B* electron is evenly emitted. With the quantified barrier hindrance effect, the average rates for the DE decay ($R_{AB}^{-6}$ asymptote in Figure 5) are indeed three orders lower than that of the averaged regular-ICD rates. Oscillations are flattened out due to the symmetry of the system. Based on electron dynamics in two-dimensional binding potentials with two-dimensional continua, in such systems a reduction of rates due to effective barriers can be expected to be less significant [17], such that in a continuum fully open in all directions, the asymptotic predictions with even a rate enhancement are supposedly fulfilled.

**Figure 7:**
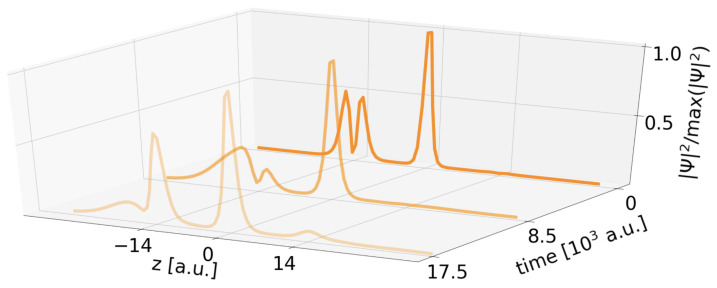
Illustration of the electron density distribution weighted to its maximal value for three propagation timesteps. The initial wavefunction has electron density corresponding to two electrons in one $A^{*}$ state and in *B* separated by $R_{AB}=14$ a.u., but no density in the other *A* site.

Having understood the overall rate trend, open questions remain on the short-distance behavior, the two additional processes CICD and exICD, and the rate outlier at $R_{AB}\approx 25.0$ a.u. To address the first, all three potential subprocesses are investigated individually, starting with the regular two-electron ICD process now in a three-QD setting with one empty QD *A* placed on the positive *z* axis. The rate is given as small blue points in Figure 5, compared to the blue-crossed rate of ICD in two QDs. Over large ranges of $R_{AB}\geq 15.5$ a.u., where the single-electron wave functions obey the asymptotic nonoverlapping condition, the rates are almost identical. They display the same oscillations known from the two-electron two-QD case as caused by the Coulomb barrier of the electron remaining in *A* and massively determining the electron emission direction [8]. However, at smaller distances the rates turn out much lower in the presence of one empty *A*-type QD. The evolution of the electron density distribution in Figure 7 can explain this observation. It shows that the electron density in the initially empty QD *A* at z=+14 a.u., which should only be a spectator, increases over time as in this nonasymptotic regime charge transfer (CT) is not excluded. Density accumulates in its lower *A* level, and is therefore no more available to ICD. This means that solely the presence of one nearby potential already slows the decay process. At the shortest $R_{AB}$, this CT effect determines the overall rate $\Gamma_{\mathrm{DE}}$ in Figure 5 as here the small blue points are matching the large dark green ones for the DE decay.

CICD was already excluded by rationalization, hence, of the other subprocess, exICD, remains for close investigation. The excited ICD process among the two outer QDs can be modeled for two electrons both in two and in three QDs. The respective graph symbols in Figure 5 are dark green crosses and small points. In general, the rates are decreasing and cover values of about $10^{-3.5}$–$10^{-6}$ a.u. that are nearly identical for large $R_{AB}\geq 16$ a.u. where CT among QDs is excluded. There, rates oscillate with a similar period as those of regular ICD of $A^{*}B$, but with a significantly lower amplitude. This goes back to the fact that the exICD electron stems from a higher energy state, has therefore a higher kinetic energy, and is conclusively much less affected by the Coulomb barrier of the remaining electron (cf. Figure 4, dark green, solid line). The $\Gamma -R_{AB}$ trend does over long ranges not follow the asymptotic $R_{AA}^{-6}$ trend, as was likewise observed for atomic clusters [23,32,34], but projections $P_{S}\left(t\right)$ on the state occupations (not shown) confirm exICD.

**Figure 8:**
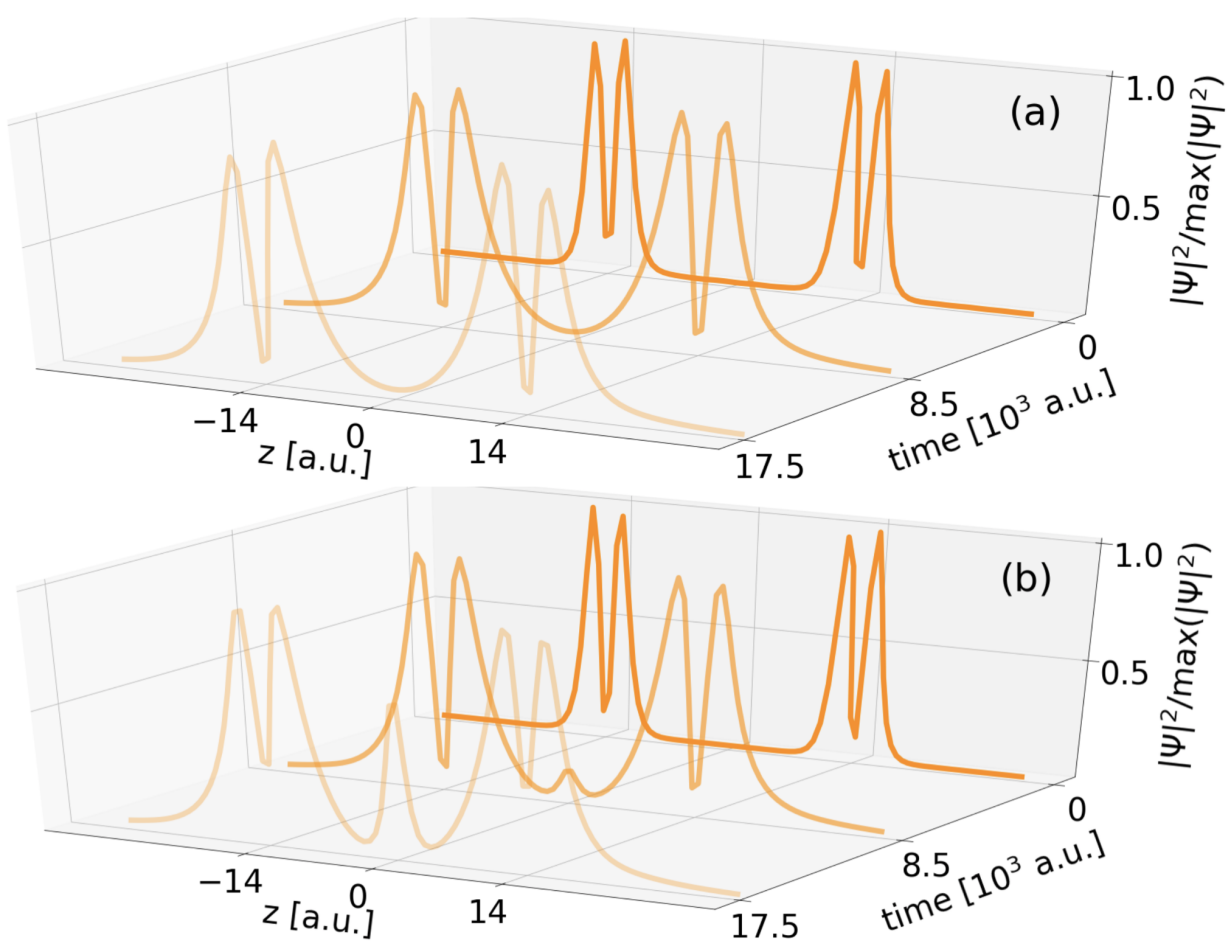
Comparison of the normalized electron density distribution of exICD of a two-electron $A^{*}A^{*}$ state in two (**a**) and three QDs (**b**) for three propagation time-steps and $R_{AA}=28$ a.u.

Another proof is the density inspection relating to the two-electron $AA^{*}$ decay in Figure 8. Panel (a) reveals that the density strictly shows occupation of the $A^{*}$ levels of the two only QDs *A*. It actually decreases over time, which is not seen due to renormalization. The sole observation is a widening of the local densities above both QDs due to the constant leak out of the continuum electron from both $A^{*}$ levels.

As for short distances $R_{AB}<16$ a.u. one finds again a discrepancy among the decay rates for two and three QDs with the difference to regular ICD that here the additional QD does not slow down the decay process as before, but actually speeds it up (small dark green dots above crosses in Figure 5). A hint for this behaviour can be gained from the electron density evolution with an additional empty *B* QD (Figure 8b). This empty well allows electron density to transfer into the *B* state and thus gives rise to regular ICD of $A^{*}B$. As ICD is obviously the faster decay pathway compared to exICD, the rate in the three-QD system is higher. Conversely, we can state that exICD is accelerated solely by the presence of one additional empty potential with a virtual *B* level in the vicinity. The process itself is not unknown. It was characterized in the context of atoms as superexchange ICD [19,20,21,22]. Here, we observe a similar rate increase for the two-electron exICD at shortening distances. As, moreover, the overall rate for three-electron DE decay at shortest distance increases, this presumably has the same origin, because the *B* level constantly gets unoccupied by regular ICD and allows for superexchange ICD.

For the larger separations $17\;a.u.\leq R_{AB}\leq 35\;a.u.$ the $R_{AB}^{-6}$ Wigner–Weisskopf prediction and the Coulomb barriers dictate the rates. In the remainder of this section, we shall explicitly analyse the dynamic properties of the processes at distances where rate outliers occur. Around $31.5\;a.u.<R_{AB}<34\;a.u.$ a few rates deviate from the asymptote. The detailed analysis of the respective densities, populations, and energies does not, however, reveal any exceptional behaviour here, so we must assume that at these small rates, the limit of numerical accuracy is reached.

The most prominent outliers toward extremely large $\Gamma_{\mathrm{DE}}$ are in the range $24.5\;a.u.<R_{AB}<27\;a.u.$ The rates at $R_{AB}=25$ and 25.5 a.u. lie almost exactly on the rate curve for regular two-electron ICD.

**Figure 9:**
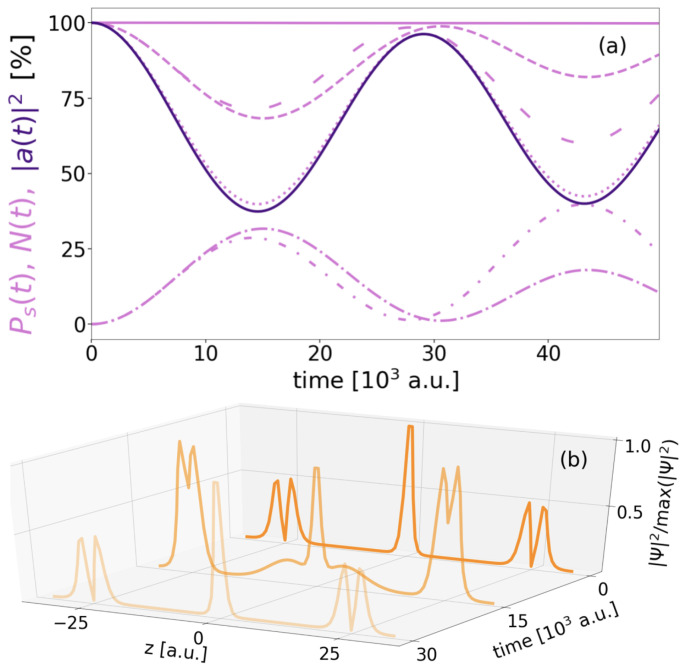
The DE decay’s single-state populations $P_{s}\left(t\right)$ (light purple, interrupted), as well as the norm N(t) (light purple solid) and the squared autocorrelation ${\left|a\left(t\right)\right|}^{2}$ (dark purple solid) as function of time for $R_{AB}=25$ a.u. in (**a**). The three individual states *s* examined are $A^{*}$ (dashed), *A* (dashed-dotted), distinguishable by tight or loose markers for right or left QD, and *B* (dotted). In (**b**) the normalized electron density distribution ${\left|\Psi \right|}^{2}/{\max(|\Psi |}^{2})$ is presented for three characteristic time steps of the propagation.

In Figure 9a, the level occupations $P_{s}\left(t\right)$ (light-purple lines, dashed for $A^{*}$, dashed-dotted for *A*, dotted for *B*), autocorrelation ${\left|a\left(t\right)\right|}^{2}$ with the initial resonance (dark purple), and the norm (light purple, solid line) are collected for $R_{AB}=25$ a.u. As uniform to all decays studied, the norm decays exponentially on the full time scale of the process. In almost all other cases (e.g., Figure 6) level populations and squared autocorrelation have followed this monotonic trend, but in the time close-up of $50\cdot 10^{3}$ a.u. in Figure 9a, they appear to oscillate strongly and periodically, the autocorrelation and the *B* population (dotted) in particular by about 50% reduction and rebuild. The population evolution of the two excited levels in the outer QDs (both dashed with different spacing) largely follow the autocorrelation in altering by half of the amount (25%), whereas the populations of the two ground states of the outer two-level QDs (dashed-dotted with different spacing) oppose. This suggests that a partial inversion of the population occurs in the respective two-level *A* sites, during which the energy is transferred to the *B* electron and exciting it. The specialty at this exclusive distance is that the *B* electron then can deexcite again. However, the three-QD system had been designed to have a single level in the *B* QD only and any excitation of the *B* electron should be into the continuum leading to disappearance of it into the CAP. Thus the question arises as to which type of state $B^{*}$ is excited by.

The electron density distributions in panel (b) for three critical time steps of the evolution shall give clarification. The initial electron density distribution shows a clear $A^{*}BA^{*}$ state. At the turning point of minimal $A^{*}$ and *B* of panel (a), i.e., after $15\cdot 10^{3}$ a.u., the density on the outer QDs is a mixture of *A* and $A^{*}$ density. Further density appears in between the *A*-sites centered around the *B*-level density peak, but filling almost all the area to the outer QDs. This indicates the excited *B* electron being trapped between the electrons in the *A* QDs. This way, the *B* electron remains in the QD region and is available to energy back-transfer to the *A* sites. And indeed, after another half period of oscillation, the initial distribution of electron density is almost regained. In the following the oscillations continue as typical for plain resonance-energy transfer [14].

**Figure 10:**
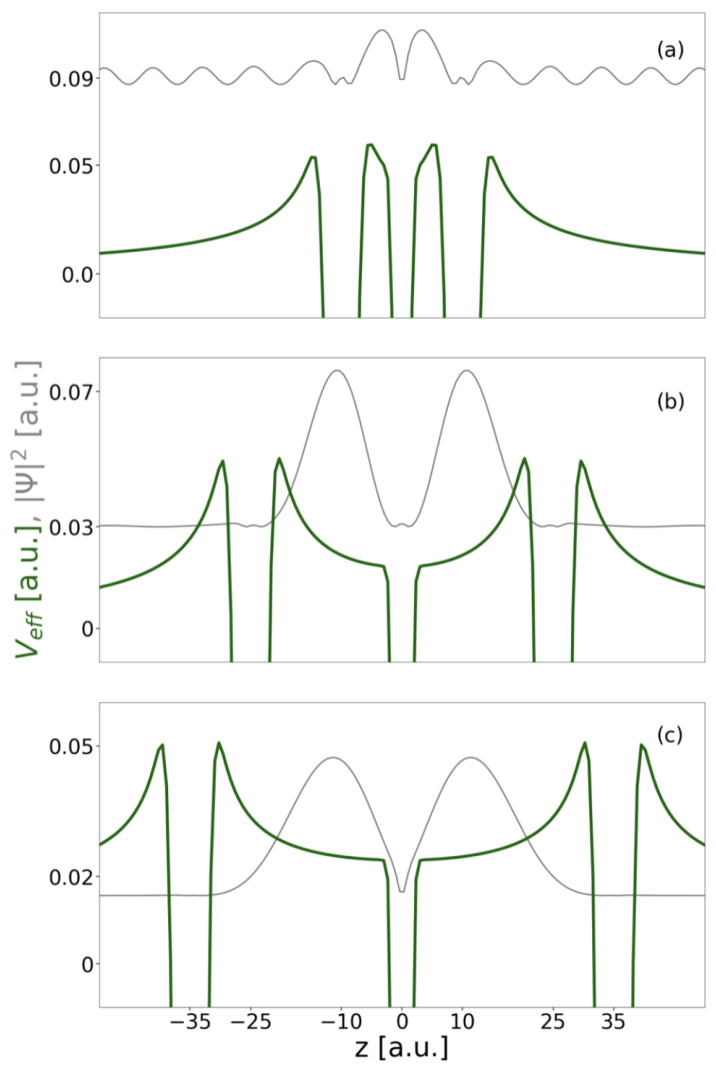
Illustration of the density ${\left|\Psi \right|}^{2}$ of the $B^{*}$ single-electron state (thin, grey line) in the effective potential of the final DE electron configuration (thick, dark green line) for (**a**) $R_{AB}=10$ a.u., (**b**) $R_{AB}=25$ a.u., and (**c**) $R_{AB}=35$ a.u.

A look at the effective potential for the final DE configuration at $R_{AB}=25$ a.u. and is associated single-electron $B^{*}$ state energy and density shall contribute to the understanding of why the process becomes so fast in this given setting (Figure 10b). The effective potential (green line) has two maxima at each side surrounding both *A* QDs. They are narrow near their peaks but widen quickly. Above the *B* side, this causes the formation of a very wide and flat potential well covering a range of approximately z∈[−20;20] a.u. Above the *B* ground state, which energetically locates in the narrow *B* potential with energy −0.459 a.u., an excited state $B^{*}$ localizes in this wide, upper well at energy 0.030 a.u. such that the excitation energy is matching the one available through $A^{*}\to A$ relaxation. This $B^{*}$ state’s electron density piles up left and right of the *B* site limited to the other side by the effective potential barriers. Its shape resembles the density contribution discovered in Figure 9b, which made the wave function available for the oscillatory energy transfer seen in Figure 9a. In addition $B^{*}$ energetically locates where the tunneling barriers are very narrow. This setting suggests that we found a shape resonance in the effective potential.

The localized $B^{*}$ state exists within a small range of distances only (where rates are high, Figure 5). If $R_{AB}$ increases, the effective potential widens and the state’s energy drops (Figure 10c). The $B^{*}$ is there facing wider and relatively high barriers, makes tunneling less likely and a shape-resonance decay thus significantly slower. On the other hand, if the distances $R_{AB}$ decrease, the energy of $B^{*}$ increases above the effective Coulomb barrier, which occurs at energies $R_{AB}=15.5$ a.u. The associated $B^{*}$ density (Figure 10a) becomes delocalized and effectively that of a true continuum state.

**Figure 11:**
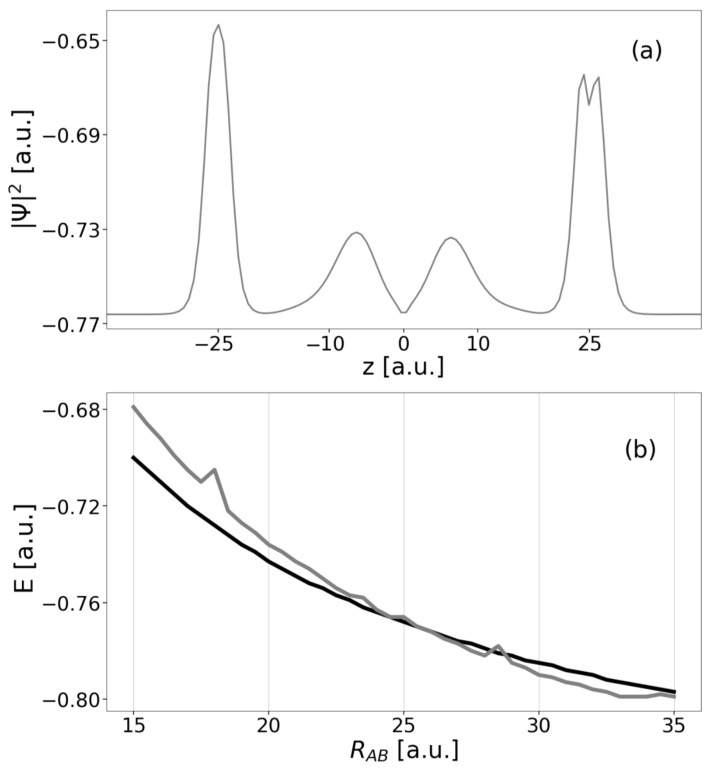
(**a**) Density ${\left|\Psi \right|}^{2}$ of the three-electron shape-resonance $AB^{*}A^{*}$ (grey) at $R_{AB}=25$ a.u. and (**b**) its state energy *E* (grey) compared to the energy of the initial electron DE state $A^{*}BA^{*}$ (black) as function of $R_{AB}$.

The existence of a resonance state alone cannot lead to a high DE-ICD rate. Energetically, the shape resonance of the effective potential must occur at the energy of the three-electron final state, i.e., at the kinetic energy of the outgoing *B* electron, and likewise of the initial state, which are themselves determined by the geometry of the three-QD system. In Figure 11a the density of the $AB^{*}A^{*}$ three-electron resonance is shown for which panel (b) presents the energy (grey) in comparison to the DE decaying state’s energy (black). Obviously, the crossing is near $R_{AB}=25$ a.u., which matches the region of the largest DE-ICD rates. In all other regions in which there is no energy matching, the distance-dependent rates $\Gamma^{\mathrm{DE}}$ align to the $R_{AB}^{-6}$ asymptote (cf. Figure 5).

Therefore, we found here a shape resonance-enhanced pathway to the decay of the $A^{*}BA^{*}$ state, which is in all other cases a Feshbach resonance decay only. The pathway can only exist in systems with a continuum confinement and is available for very few energy settings. During the shape resonance-enhanced decay, the electronic configuration belonging to the shape resonance is reached quickly (within $15\cdot 10^{3}$ a.u., Figure 9). It then decays efficiently into the final states $A^{*}B^{+}A$ and $AB^{+}A^{*}$, as shape resonances always decay faster than two-electron Feshbach resonances (e.g. the ICD initial state) [8].

#### 4.2.2. Dynamics of the Singly Excited Resonance

In the following, we will focus on the dynamical processes of the SE resonance $A^{*}BA$, which include regular ICD among QDs *A* and *B*, as well as the Förster-like resonance energy transfer among the two outer QDs *A* (cf. Figure 1, bottom), where the latter, however, should not lead to a decay in competition with ICD. The expectation formulated for the decay is straightforwardly $\Gamma_{\mathrm{SE}}=\Gamma_{\mathrm{SE}}^{ICD}$. To recall, the SE resonance is a superposition of the two symmetry-equivalent eigenstates with a single excitation of either of the outer QDs, the left ($A^{*}BA$) or right ($ABA^{*}$). Therefore, every quantitative analysis (e.g., rates) is made according to this superposition.

Figure 5 shows $\Gamma_{\mathrm{SE}}$ as light green bold dots, revealing it to be the lowest overall rate at most inter-QD separations. The $\Gamma_{\mathrm{SE}}$-$R_{AB}$ graph divides into two ranges with different decay behavior. The rates at the short-distance range $R_{AB}\leq 14.0$ a.u., where the asymptotic equations are not valid, decrease steeply and are nearly equal to the DE rates. For larger distances $R_{AB}\geq 14$ a.u. the SE rates drop below the DE rates and then follow the $R_{AB}^{-6}$ asymptote. Overall outliers toward lower rates in both zones are the process at $R_{AB}=11$ a.u. and those from $R_{AB}>30$ a.u. The latter very low rates drop significantly below the asymptote and are artifact of the numerical limits of the calculations.

To understand the decay behavior, we can benefit from our investigations of DE processes. In SE processes, neither accelerating nor decelerating short-range CT effects can be observed. An acceleration effect would require an enhancement of the population of an $A^{*}B$ state. In the DE case, this was obtained through CT of the $A^{*}$ electron from the other site to *B*. Here, the corresponding tunneling would have to be from the lower *A* level with narrow density, which is energetically and in terms of overlap not favorable. Compared to the DE resonance, the SE resonance energy is almost twice as low (Figure 3), hence there are fewer ionization channels below. In the long-distance region of the $\Gamma_{\mathrm{SE}}$-$R_{AB}$ graph, SE dynamics does, like DE dynamics, follow the asymptote $R_{AB}^{-6}$ without drastic rate oscillations as known from the regular ICD. The reason for this flatness is the symmetry of the effective barrier forming the *B* electron confinement. The rates are even one order slower as it has a significantly higher effective barrier to tunnel (cf. Figure 4).

**Figure 12:**
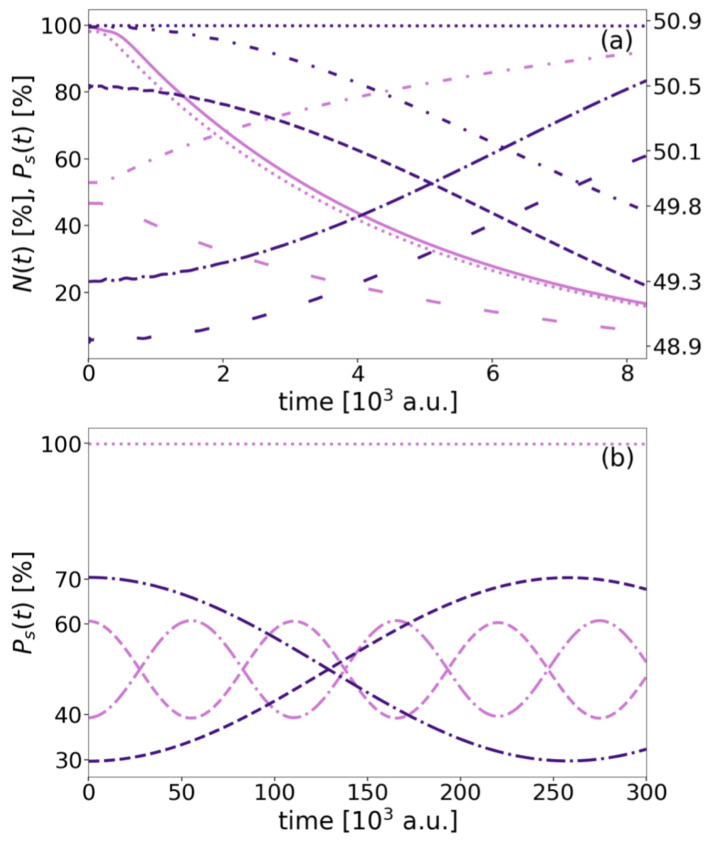
Single-state populations $P_{S}\left(t\right)$ (interrupted lines) and norm N(t) (solid) in % as function of the propagation time for the SE decay. In (**a**) the left ordinate and light purple lines belong to $R_{AB}=10$ a.u., whereas dark purple lines and the right ordinate correspond to $R_{AB}=13$ a.u. In (**b**) projections corresponding to $R_{AB}=21$ a.u. (34. a.u.) are indicated in light (dark) purple. The five individual states examined are $A^{*}$ (dashed), *A* (dashed-dotted), each tight or loosely for right or left QD in (**a**), and *B* (dotted).

As there are neither marked outliers in the behavior of Γ, nor does CT at short distances apply, the last analysis is directed to the observation of ET among the outer QDs as a potential side process to ICD. The norm and level populations of the three-electron SE dynamics are therefore compared for four representative distances (Figure 12). The initial wave functions for the analyses are obtained from the improved block relaxation and mostly represent neither a complete superposition nor a pure eigenstate of $A^{*}B^{+}A$ and $AB^{+}A^{*}$, but actually their linear combinations. An even superposition, where the right QD is occupied in the *A* (dashed-dotted) and the $A^{*}$ (dashed) level each by 50% arises for the case $R_{AB}=10$ a.u. (Figure 12a, light purple lines). An identical occupation holds for the left QD (not shown). Over time, the population of the $A^{*}$ levels reduces toward 0%, i.e., the *A* levels’ occupation inverts. At the same time the *B* population (dotted) and the overall norm (solid line) both decrease exponentially from 100% according to $\Gamma^{\mathrm{SE}}$ as depicted in Figure 5. This is in principle the behavior as expected for a regular two-electron two-QD ICD process [56], but first traces of ET among both *A* QDs are evident from the numerical data.

This gets more pronounced and even visible from the propagation, when going to the larger distances, $R_{AB}=13$ a.u. (Figure 12a, dark purple), $R_{AB}=21$ a.u. and $R_{AB}=34$ a.u. (light and dark purple in panel (b) with a longer observation time), where the dominance of ICD decreases. The decay becomes inherently slower, such that the *B* population and the norm remain majorly at their initial 100% in the displayed time window. In these scenarios, the level populations of the outer QDs display dominantly Förster-like ET dynamics by periodically inverting between $A^{*}$ and *A* of the coupled dipoles over time, and overall $A^{*}A⇌AA^{*}$. For $R_{AB}=13$ a.u. the full inversion is beyond the displayed data in (a), while the inversion or transfer time is $56\cdot 10^{3}$ a.u. for $R_{AB}=21$ a.u. and $257\cdot 10^{3}$ for $R_{AB}=34$ a.u. By using similar data for further distances we observe the transfer rate, the inverse of the transfer time, decrease with increasing $R_{AB}$. This is not surprising, as energy transfer processes also depend on Coulomb interaction (Equation (4)). Here, ET rates follow an $R_{AB}^{-3}$ trend. This indicates that not all assumptions of a dipole–dipole transition are valid in the present system, because the short-range ET in atomic and molecular systems of typical electron excitation energies is supposed to be the coupling of dipole transitions leading to the well-known $R_{AB}^{-6}$ dependence (Section 2.1). However, in other studies it was shown that the *R* dependence is not trivially predictable in significantly altered geometries and systems [14]. There is intermediate-range transfer with an $R_{AB}^{-4}$ dependence and long-range energy transfer with a $R_{AB}^{-2}$ rate dependence depending on the relation of size of the ET partners and the wave length of the transferred virtual photon. Besides the plain theory, distance dependencies of $R_{AB}^{-3}$ and $R_{AB}^{-5}$ have also been reported [63].

The absolute rates for this ET are all in the range of $10^{-5}$ a.u. for the wide range of distances given. Explicitly, they are $4.78\cdot 10^{-5}$ a.u. $>\Gamma_{\mathrm{SE}}^{\mathrm{ET}}>0.39\cdot 10^{-5}$ a.u. within 15.0 a.u. $<R_{AB}<34.0$ a.u. If comparing the rates with $\Gamma_{\mathrm{SE}}=\Gamma_{\mathrm{SE}}^{\mathrm{ICD}}$ in Figure 5, their crossing occurs near $R_{AB}$ = 11.0–13.0 a.u., which matches the distance from which on CT of populations is overlaying ICD (Figure 12).

## 5. Conclusions

This paper comprises the study of the interparticle Coulombic decay process in an array of three linearly aligned binding sites with two virtual-photon donors *A* at the edges and an electron emitter *B* in the center. This ABA system design was chosen to provide a delineation of information to previously studied three-site ICD processes. To complement this, we investigate two possible excitation scenarios. In the SE scenario, only one photon donor is initially excited, whereas in the DE process, both *A* sites are. This work’s investigations explore the electronic structure of the model system and, moreover, give a detailed description of the dynamics of three electrons in three linearly aligned QDs.

In a rationalization of possible subprocesses along with the formulation of their Wigner–Weisskopf rates, we analytically confirm predictions of at least a rate doubling with doubling of the number of photon emitters in agreement with previous findings. However, because this rate doubling is not confirmed by highly correlated electron dynamics, but rather a strong rate decrease is found, all subprocesses are disentangled and studied individually. These are namely two-electron regular ICD for both initial states, for the DE case additionally excited ICD among only to excited photon emitters and collective ICD of three electrons, and for the SE case resonance energy transfer among the outer sites.

The breakdown into the individual subprocesses in conjunction with geometrical and energetic analyses revealed that a third, empty, site can affect the rates due to its ability to bind the electronic wave packet of a nearby neighboring site, which can in cases enhance (by superexchange ICD), and in others decrease the overall rates, depending on whether the electrons Coulomb interact more or less as a result of such charge transfer. Furthermore, linking information on the evolution of state energies and effective Coulomb barriers with inter-QD distance to dynamic quantities such as decay rates and population analyses provides insight into rate evolutions and relative speed of subprocesses.

For longer distances, a significant slowdown of the three-electron dynamics occurs compared to that of two electrons. We attribute this effect to the effective barriers hindering the ICD electron in the one-dimensional continuum to travel to any direction. In the SE case, those barriers are higher; hence, a generally more significant rate slowing is observed. This means that in the asymptotic regime, the decay of the SE or DE resonance would be overlaid by phonon-mediated dissipation [48] or radiative decay [8], which both have rates of about $10^{-6}$ a.u.

In the DE case, at a certain distance between sites, a synergistic effect of continuum confinement, energy levels, and Coulomb interaction emerges a shape-resonance pathway with an extraordinarily large rate. Here the *B* electron is initially excited into a localized, but nonetheless extended wave packet above the *B* site, from where it can decay quickly into the continuum.

## Figures and Tables

**Table 1:** Energies in a.u. of single- ($E_{1e}$) and three-electron states ($E_{3e}$), in the latter case for the minimum and maximum distance, $R_{AA}^{min}=20$ a.u. and $R_{AA}^{max}=70$ a.u., respectively.

$E_{1e}$		$E_{3e}$	$R_{\mathrm{AA}}^{\min}$	$R_{\mathrm{AA}}^{\max}$
$E_{A^{*}}$	−0.196	$E_{A^{*}BA^{*}}$	−0.613	−0.797
$E_{B}$	−0.477	$E_{A^{*}BA}$	−1.113	−1.295
$E_{A}$	−0.693	$E_{ABA}$	−1.613	−1.793

**Table 2:** Barrier energies $E_{CB}^{f}$ and minimal distances $R_{AB}^{f}$ from which the kinetic energy of electron *B* (for exICD *A*) drops below $E_{CB}^{f}$ are given for the effective Coulomb barriers resulting from final *f* electron configuration of the DE, SE, regular ICD, and exICD process.

	DE	SE	ICD	exICD
$E_{CB}^{f}$ (a.u.)	0.054	0.070	0.056	0.152
$R_{AB}^{f}$ (a.u.)	17.0	25.0	15.5	>35.0

## Data Availability

Data is available upon request from the authors.
